# Evaluating machine learning pipelines for multimodal neuroimaging in small cohorts: an ALS case study

**DOI:** 10.3389/fninf.2025.1568116

**Published:** 2025-06-13

**Authors:** Shailesh Appukuttan, Aude-Marie Grapperon, Mounir Mohamed El Mendili, Hugo Dary, Maxime Guye, Annie Verschueren, Jean-Philippe Ranjeva, Shahram Attarian, Wafaa Zaaraoui, Matthieu Gilson

**Affiliations:** ^1^Aix Marseille Univ, CNRS, CRMBM, Marseille, France; ^2^Aix Marseille Univ, CNRS, INT, Marseille, France; ^3^APHM, Hopital de la Timone, Referral Centre for Neuromuscular Diseases and ALS, Marseille, France

**Keywords:** amyotrophic lateral sclerosis, machine learning, multimodal MRI, small cohort, classification, pipeline optimization

## Abstract

Advancements in machine learning hold great promise for the analysis of multimodal neuroimaging data. They can help identify biomarkers and improve diagnosis for various neurological disorders. However, the application of such techniques for rare and heterogeneous diseases remains challenging due to small-cohorts available for acquiring data. Efforts are therefore commonly directed toward improving the classification models, in an effort to optimize outcomes given the limited data. In this study, we systematically evaluated the impact of various machine learning pipeline configurations, including scaling methods, feature selection, dimensionality reduction, and hyperparameter optimization. The efficacy of such components in the pipeline was evaluated on classification performance using multimodal MRI data from a cohort of 16 ALS patients and 14 healthy controls. Our findings reveal that, while certain pipeline components, such as subject-wise feature normalization, help improve classification outcomes, the overall influence of pipeline refinements on performance is modest. Feature selection and dimensionality reduction steps were found to have limited utility, and the choice of hyperparameter optimization strategies produced only marginal gains. Our results suggest that, for small-cohort studies, the emphasis should shift from extensive tuning of these pipelines to addressing data-related limitations, such as progressively expanding cohort size, integrating additional modalities, and maximizing the information extracted from existing datasets. This study provides a methodological framework to guide future research and emphasizes the need for dataset enrichment to improve clinical utility.

## 1 Introduction

By allowing for the non-invasive visualization of anatomical and functional problems in the brain, medical imaging has transformed our understanding of neurological illnesses. Magnetic Resonance Imaging (MRI) is one such imaging technique that has become an indispensable tool for understanding the structural and functional basis of neurological disorders (Elias-Jones et al., 1990; Weiner, 2024; Shevchenko et al., 2025; Grapperon et al., 2025). They have been widely employed in the detection of diseases and their diagnosis, as well as in the monitoring of treatment plans. Brain imaging, in particular, plays a crucial role as the brain is one of the most complex organs in the human body and is involved in a wide range of neuropathologies, including neurological and psychiatric disorders. Despite these capabilities, the interpretation of MRI data remains highly complex in the clinical context, especially for multifactorial and heterogeneous conditions such as Multiple Sclerosis (MS), Amyotrophic Lateral Sclerosis (ALS), Epilepsy and Parkinson's disease (Fox et al., 2011; Zejlon et al., 2022; El Mendili et al., 2023). These diseases often remain undetected until they reach advanced stages, leading to delayed interventions and suboptimal outcomes.

Advancements in Artificial Intelligence (AI) hold immense possibilities for the field of medical imaging, offering the potential to unravel subtle imaging patterns beyond human perception (Yousefirizi et al., 2022). These technologies not only improve diagnostic accuracy, but also significantly reduce the time required for data analysis and interpretation. The integration of AI into neuroimaging workflows is particularly promising in the context of multimodal MRI data, where multiple complementary modalities can be leveraged to improve prediction accuracy when combined with clinical information. AI techniques, particularly Machine Learning (ML) and Deep Learning (DL), hold immense promise for effectively integrating such multimodal, multiparametric data to identify novel biomarkers (Wang et al., 2018; Mirabnahrazam et al., 2022). Such biomarkers can aid in early diagnosis, prognostication, and treatment response monitoring for neurodegenerative diseases.

There still exist major challenges to the application and optimal utilization of AI-based approaches for neuroimaging in a clinical context. The need for access to large datasets to train these ML/DL models is one such challenge. AI models require large, diverse datasets to generalize effectively (Lin et al., 2021; Grollemund et al., 2019). However, the study of neurodegenerative disorders through medical imaging frequently involves datasets that are quite limited and notably imbalanced. This can often be due to the nature of the rare disease, such as ALS (Zhang et al., 2011; Tilsley et al., 2024), and further amplified when they are very debilitating, thus restricting the number of patients that are physically capable of participating in these studies. Acquiring vast amounts of data in such scenarios is often impossible, as the state of the patients often deteriorates rapidly, contributing to significant dropout rates (Drory et al., 2001; Ashworth et al., 2012). In such cases, significant efforts are often directed toward optimization of the machine learning pipelines in order to improve the overall outcomes.

In this study, we aim to systematically investigate the design and optimization of machine learning pipelines for analyzing multimodal neuroimaging data from a small cohort of ALS patients. Specifically, we explore how various preprocessing techniques, feature selection methods, and model architectures affect performance under data-constrained conditions. Furthermore, we compare the performance gains achieved through fine-tuning of the ML pipeline with those obtained by enriching the input dataset. We also highlight the useful insights that ML can provide for small datasets, in terms of guiding further data collection by capturing synergistic effects between modalities. In doing so, we seek to establish a methodological framework that balances computational rigor with clinical applicability, ultimately contributing to the broader understanding of ALS and the development of ML tools for rare neurological conditions.

## 2 Materials and methods

### 2.1 Cohort description

The study included a total of 30 participants, comprising 16 individuals diagnosed with ALS (6 females, 10 males; mean age: 61.7 years, SD: 13.1 years) and 14 healthy controls (8 females, 6 males; mean age: 55.1 years, SD: 6.9 years). Each participant underwent multimodal imaging using a 7T MRI scanner, generating 270 parametric datasets across all subjects. The ALS cohort was recruited from the ALS reference center at our university hospital, with diagnoses confirmed based on the revised El Escorial criteria (Brooks et al., 2000). All participants in this group were free of other neurological conditions and screened to rule out frontotemporal dementia following international consensus criteria (Rascovsky et al., 2011). Clinical assessments for ALS patients were conducted immediately after their MRI scans, including scoring on the Revised Amyotrophic Lateral Sclerosis Functional Rating Scale (ALSFRS-R) (Cedarbaum et al., 1999). Disease progression rates were quantified using the ALSFRS-R slope, calculated as (48 - ALSFRS-R score) divided by the disease duration (in months). Based on this metric, patients were categorized into slow or fast progressors, with the median ALSFRS-R slope value of 0.62/month chosen as the threshold for classification (Labra et al., 2016).

The healthy control group consisted of individuals with no prior history of neurological or psychiatric disorders and normal findings during clinical examinations. The limited cohort size is a reflection of various factors: the rarity of the disease, the nature of the disease, and the complexity of acquiring high-quality multimodal MRI data (Zhang et al., 2011; Tilsley et al., 2024). This sample size is consistent with prior neuroimaging studies in ALS (Zhang et al., 2011; Atassi et al., 2017; Barry et al., 2021; El Mendili et al., 2023), which have similarly aimed to extract biomarkers for ALS or evaluated group-level trends under data-constrained conditions. While the small size of the cohort imposes limitations on statistical power and generalizability, it provides a realistic setting to assess the reliability and stability of ML pipeline components in rare disease contexts.

Ethical approval for this study was granted by the Comité de Protection des Personnes Sud-Méditerranée 1, in accordance with the Declaration of Helsinki. All participants provided written informed consent prior to enrollment.

### 2.2 Data acquisition and pre-processing

MRI data acquisition was performed using a 7 Tesla Magnetom whole-body MR system (Siemens, Erlangen, Germany). Proton imaging utilized a 32Rx/1Tx 1H head coil, and the protocol included a high-resolution 3D T1-weighted Magnetization Prepared 2 Rapid Acquisition Gradient Echoes (MP2RAGE) sequence (TR = 5,000 ms, TE = 3 ms, TI1 = 900 ms, TI2 = 2750 ms, 256 slices, isotropic resolution of 0.6 mm, GRAPPA acceleration factor = 3, acquisition time = 10 min 12 s), a 2D magnetization-prepared turbo FLASH $B_{1}^{+}$ mapping sequence (TR = 2,000 ms, TE = 14 ms, 14 transverse slices with a thickness of 5 mm, in-plane resolution = 3.9 mm × 3.9 mm, acquisition time = 2 min 14 s) and two diffusion-weighted echo planar imaging sequences, acquired with 80 diffusion encoding directions (anterior-posterior and posterior-anterior) and 13 b0 volumes for diffusion tensor imaging (DTI), with b-values of 0, 1,000, and 2,000 *s*/*mm*^2^ (TR = 6,000 ms, TE = 79.2 ms, 120 transverse slices, isotropic resolution of 1.13 mm, MultiBand factor = 3, acquisition time = 10 min 42 s for each encoding phase).

Sodium imaging was conducted with a dual-tuned ^23^Na/1H 1Tx/1Rx head coil (QED), using a multi-echo density-adapted 3D projection reconstruction pulse sequence (TR = 120 ms, 5,000 spokes, 384 radial samples/spoke, isotropic resolution of 3 mm, 24 TEs spanning 0.20–70.78 ms in three sets of 8 echoes each, acquisition time = 3 × 10 min). Six cylindrical tubes with sodium concentrations ranging from 25 to 100 mM in 2% agar, were placed within the field of view near the participant's head, to serve as external quantitative references.

The MP2RAGE sequence provided a T1-weighted (UNI) image with reduced reception bias fields and enabled the generation of quantitative T1 (qT1) maps. These qT1 maps were corrected for $B_{1}^{+}$ inhomogeneity, denoised, and skull-stripped before further processing. Diffusion-weighted images underwent preprocessing steps, including denoising, Gibbs-ringing artifact removal, and corrections for B1 field inhomogeneity, susceptibility distortions, eddy current effects, and head motion. From these data, maps of Fractional Anisotropy (FA), Mean Diffusivity (MD), Axial Diffusivity (AD), and Radial Diffusivity (RD) were derived. Sodium imaging data were reconstructed and denoised using a multi-echo approach, and motion correction was applied. Quantitative maps were computed for total sodium concentration (TSC), T2*short (T2s), T2*long (T2l), and the sodium signal fraction (*f*_*Na*_), representing the short component of the biexponential signal decay.

T1-weighted images (T1w) were bias field-corrected using the N4 algorithm (Tustison et al., 2010) and aligned to the AC-PC plane via rigid registration to the MNI152 template. These images were parcellated using FreeSurfer (v7.4.1) (Fischl, 2012) with the Destrieux atlas (Destrieux et al., 2010), generating two parcellation schemes: (i) 187 regions of interest (ROIs), primarily cortical and subcortical areas, along with five macroscopic white matter regions, and (ii) 332 ROIs, which included 150 additional white matter-specific regions. Volumetric ROI masks were initially produced in FreeSurfer's intrinsic *fsaverage* space (equivalent to MNI305) and subsequently transformed into each subject's native space. For simplicity, the two parcellation schemes are referred to as GM (187 ROIs) and GM+WM (332 ROIs). The GM+WM parcellation was adopted for all evaluations, unless indicated otherwise.

Quantitative imaging maps (qT1, FA, MD, AD, RD, TSC, T2s, T2l, and *f*_*Na*_) were coregistered with the denoised T1w images using the ANTs library (Avants et al., 2009). The ROI masks, now aligned to each subject's native space, were applied to all imaging maps. Mean voxel values were calculated within each ROI for each quantitative map, producing matrices of dimensions: (i) 187 ROIs × 30 subjects, and (ii) 332 ROIs × 30 subjects. Multimodal analyses were performed by concatenating the individual quantitative maps.

### 2.3 Machine learning workflow

In view of the limited size of the ALS dataset, traditional ML approaches were adopted in this study, as opposed to more advanced and powerful DL techniques that require large amounts of data for model training. Several studies have reported that traditional ML techniques can perform as well as, or even better than, DL approaches when dealing with small datasets (Nanni et al., 2020; Islam and Khanam, 2024). To rigorously evaluate model performance and tuning, we adopted a nested cross-validation approach. The outer loop employed Leave-One-Out Cross-Validation (LOOCV), where each subject was iteratively held out for testing. Within each outer fold, hyperparameter tuning (when included in the pipeline) and preprocessing were carried out using 5-fold stratified cross-validation on the remaining subjects. All preprocessing steps—including scaling, feature selection, and dimensionality reduction—were confined to the training folds to prevent data leakage. This nested strategy ensures an unbiased estimation of model performance by separating model evaluation from model selection. It is particularly valuable in settings with limited or imbalanced data, as it reduces the risk of overfitting to the training data and provides a more reliable estimate of how the model will generalize to unseen subjects.

For the first round of evaluations, the pipeline began with row-wise (i.e., subject-wise) scaling for each imaging map, followed by column-wise (i.e., feature-wise) scaling within each map; in both cases by removing the mean and scaling to unit variance using the StandardScaler module of the sklearn library. The column-wise scaling was performed separately within the training and validation folds to prevent data leakage and ensure that scaling was based solely on the training data. This approach preserved the integrity of the cross-validation procedure by ensuring the model did not inadvertently gain access to any information from the validation set, thereby avoiding overfitting and ensuring a more reliable assessment of model performance. A large number of classifiers were initially explored, largely in their default configurations. Those that demonstrated promising outcomes were shortlisted for further evaluation.

In the second stage, the classifiers were kept constant while various other steps were systematically varied to assess their individual and combined impact on model performance. Specifically, we evaluated different configurations of data scaling, feature selection, and dimensionality reduction.

For scaling, we tested three options: no scaling, standardization (using StandardScaler, which centers features to zero mean and unit variance), and normalization [using MinMaxScaler, which rescales features to a (0, 1) range]. These choices were motivated by the fact that many ML algorithms are sensitive to feature magnitude and distribution. By comparing performance across these variations, we aimed to understand how such preprocessing decisions influence downstream classification accuracy.

When applied, feature selection was performed using either SelectKBest or Recursive Feature Elimination (RFE). For SelectKBest, features were scored using the ANOVA F-value (f_classif) as the scoring function, which evaluates the relationship between each feature and the target class in a univariate manner. RFE, which iteratively removes the least important features, was implemented using a linear-kernel Support Vector Regressor (SVR) as the estimator. This model was chosen for its ability to rank features based on the magnitude of regression weights, allowing recursive elimination of the least informative features. We explored a range of feature counts for both these methods, and also assessed the performance when no feature selection was involved.

Dimensionality reduction was conducted using either Principal Component Analysis (PCA) or Linear Discriminant Analysis (LDA). PCA was applied in an unsupervised fashion to retain the main components explaining the variance within the training fold. Linear Discriminant Analysis (LDA) was applied as a supervised dimensionality reduction technique that seeks to maximize class separability by projecting the data onto a lower-dimensional space.

The final stage of evaluations involved keeping the above components of the pipeline constant, chosen based on the best performers, while tuning the hyperparameters of the classifiers. Hyperparameter optimization was explored using three distinct techniques: (i) grid search, (ii) random search, and (iii) Bayesian optimization. All three were implemented within the inner loop of a nested cross-validation framework, using their respective modules in the sklearn library. The details of the search spaces employed for the various classifiers are specified in Supplementary Table S1 of the Supplementary material. These ranges were selected based on commonly used defaults and prior experience in similar neuroimaging ML studies, and were intended to provide a balanced search space broad enough to capture performance-relevant variability, yet constrained to avoid overfitting, in view of the small sample size. Furthermore, to ensure a fair comparison, the search spaces were matched across all three methods and the total number of parameter combinations tested was kept constant. The latter was achieved by evaluating the total number of possible combinations for the grid search space, and then enforcing this value as the maximum permissible iterations for both random and Bayesian searches. A schematic representation of the ML pipeline is provided in Supplementary Figure S1 of the Supplementary material.

All code was implemented in Python, with the machine learning experiments executed on the computing nodes of the institute's HPC infrastructure (Mesocentre). The jobs were run on Dell PowerEdge C6420 nodes, each equipped with 32 CPU cores, powered by Intel^®^ Xeon^®^ Gold 6142 (Sky Lake) processors running at 2.6 GHz. Each job was allocated one node, utilizing all 32 CPU cores, and the multiprocessing functionality was implemented in Python using the multiprocessing module to efficiently parallelize the workload. Jobs were submitted using the SLURM job scheduler. Jobs typically took 3x longer when run locally on a workstation, as compared to the HPC system; see section 4 of the Supplementary material for more information.

### 2.4 Performance evaluation

A variety of metrics are available for evaluating ML model performance for classification tasks. These include accuracy, precision, recall, F1 score and AUC-ROC. For multi-class classification, there exist many more related metrics. In this study, we opted to employ the classification metric as the principal metric for comparing between various configuration of the ML pipeline. This was largely driven by the intuitive and straightforward measurement of this statistic, and applies easily for both the 2-class and 3-class evaluations undertaken here. Also, the primary aim of this study was to compare the effects of various changes to the ML pipeline, rather than to focus on specific class-level performance. Classification accuracy effectively captures the aggregate performance of the model across all classes, making it a suitable choice for high-level evaluation and comparison. Furthermore, the LOOCV approach adopted in the pipeline evaluates the model's ability to generalize by iteratively training on all but one sample and testing on the held-out sample. Accuracy aligns naturally with this validation method by providing a single aggregate score that summarizes performance across all cross-validation folds.

In addition to the accuracy of the model, the robustness and reliability of its predictions is also an important factor. This is often assessed by comparing the classification accuracy to a baseline or chance-level distribution obtained through permutation testing. In our study, each classification task was repeated 50 times with randomly shuffled labels, effectively breaking the association between the input data and their corresponding labels, to generate a distribution of chance-level accuracies. Since this distribution was approximately normal, we were able to calculate a Z-score to quantify how much the model's actual accuracy deviated from the chance-level performance.

Furthermore, to assess the statistical significance of performance differences between our various ML pipeline configurations, we conducted Wilcoxon signed-rank tests by comparing them to their respective baseline counterparts, while discarding all zero-differences (i.e. *wilcox* method). These analyses confirmed that while some improvements are statistically significant, many configurations are not. Moreover, in several cases with statistical significance, the effect size indicated a deterioration in the performance.

## 3 Results

Below, we shall discuss the impact of the various components of the ML pipeline on the classification outcomes. This is presented in three stages as outlined earlier. We then also present an overview of how the nature of the input dataset influences the performance. Each evaluation of the ML pipeline was executed as a single SLURM job with three classification sub-tasks:

- **2-class (controls vs. patients)**: the model is required to differentiate between healthy controls and patients with ALS.
- **3-class (controls vs. slow ALS vs. fast ALS)**: the model is required to differentiate between healthy controls and patients with slow and fast progressing ALS.
- **slow ALS vs. fast ALS (SvF)**: the model is required to differentiate between patients with slow and fast progressing ALS.

With an eye on their clinical relevance, we prioritize the 3-class classification outcomes, as they provide the most actionable insights for patient care. This is followed by the Slow vs. Fast classification, which enables distinguishing between clinical states that determine prognosis. While less informative for clinical actions, the 2-class performance holds value in understanding the underlying brain alterations associated with pathology, thereby offering a broader understanding of disease mechanisms.

### 3.1 Preliminary evaluation of classifiers

The field of machine learning (ML) offers a vast array of classifiers, each with unique strengths and limitations. Most classifiers offer access to various internal parameters that allow us to customize their operation. Given this diversity, it is not practical to evaluate each such classifier, and their innumerable variants, comprehensively. In an effort to quickly identify which classifiers are promising for our study, we focused on 12 widely employed and popular classifiers. The classifiers chosen were: K-Nearest Neighbors (KNN), Light Gradient Boosting Machine (LGBM), Multinomial Logistic Regression with Elastic Net Regularization (MLR_ENET), Multinomial Logistic Regression with L1 Regularization (MLR_L1), Multinomial Logistic Regression with L2 Regularization (MLR_L2), Multi-Layer Perceptron (MLP), Polynomial Kernel Support Vector Machine (SVM_POLY), Radial Basis Function Support Vector Machine (SVM_RBF), Sigmoid Kernel Support Vector Machine (SVM_SIG), Linear Support Vector Machine (SVM_LIN), Random Forest (RF), and Extreme Gradient Boosting (XGB).

**Figure 1:**
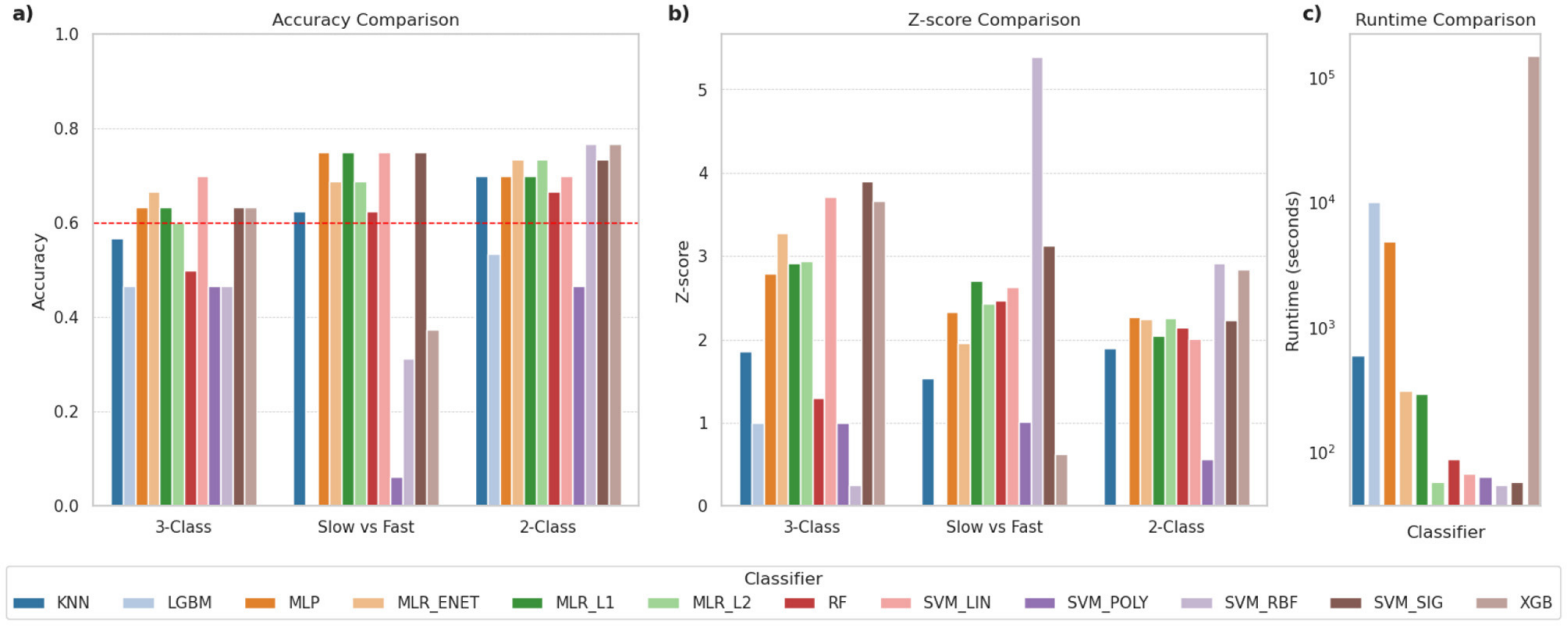
Comparison of classification performance for various classifiers. The panels compare **(a)** classification accuracy, **(b)** Z-scores indicating robustness of predictions, and **(c)** time required for execution (in seconds), using log scale. The dashed red-line in **(a)** indicates 60% accuracy and was used as a threshold to shortlist classifiers for further evaluations.

For the initial phase of evaluation, these classifiers were used primarily in their default configurations, i.e., without any attempts to optimize their hyperparameters, to establish a baseline performance. As mentioned earlier, each evaluation consisted of three classification tasks (i.e., 3-class, 2-class, SvF). The performance for each task was evaluated by means of the accuracy score and the corresponding Z-score value, thereby comparing the reported accuracy against chance level predictions. Figure 1 shows the comparison of the classification performance of the various models across the three tasks. The SVM_LIN and SVM_SIG classifiers performed well across all classification tasks, alongside the three MLR-based classifiers. We notice that certain classifiers such as LGBM and SVM_POLY performed very poorly in general, while some of the other classifiers show lower accuracies on one or more of the other tasks. In addition, Figure 1c, using log-scale, shows that LGBM, MLP and XGB classifiers took significantly more time, and thereby computational resources, to complete these tasks.

In order to shortlist a subset of these classifiers for further evaluations, we applied a threshold of 60% classification accuracy across the three tasks. This is indicated by the red dashed line in Figure 1a. Based on this criterion, the following classifiers met the threshold: MLP, MLR_ENET, MLR_L1, MLR_L2, SVM_LIN and SVM_SIG. However, due to the significant computational demands of the MLP classifier, it was deemed difficult to accommodate it in the next stage, which included extensive ML pipeline configuration explorations. The MLP classifier was therefore dropped from this next stage, but was reintroduced in the third stage focused on hyperparameter optimization, where other pipeline components were kept constant. In its place, we selected the RF classifier in view of its versatility across various datasets, and its suitability as a benchmark for tree-based ensemble methods.

### 3.2 Pipeline refinement

Here, we explored the effects of modifying the ML pipeline on classification outcomes by systematically varying key pipeline components. This included altering the row and column scaling methods, and incorporating additional steps in the pipeline, such as performing feature selection and dimensionality reduction. This involved evaluating 297 combinations for each of the six classifiers we shortlisted from the previous stage, for a total of 1,782 trials. The objective was to optimize the performance of the pipeline by identifying the most promising configurations for our classification tasks.

**Figure 2:**
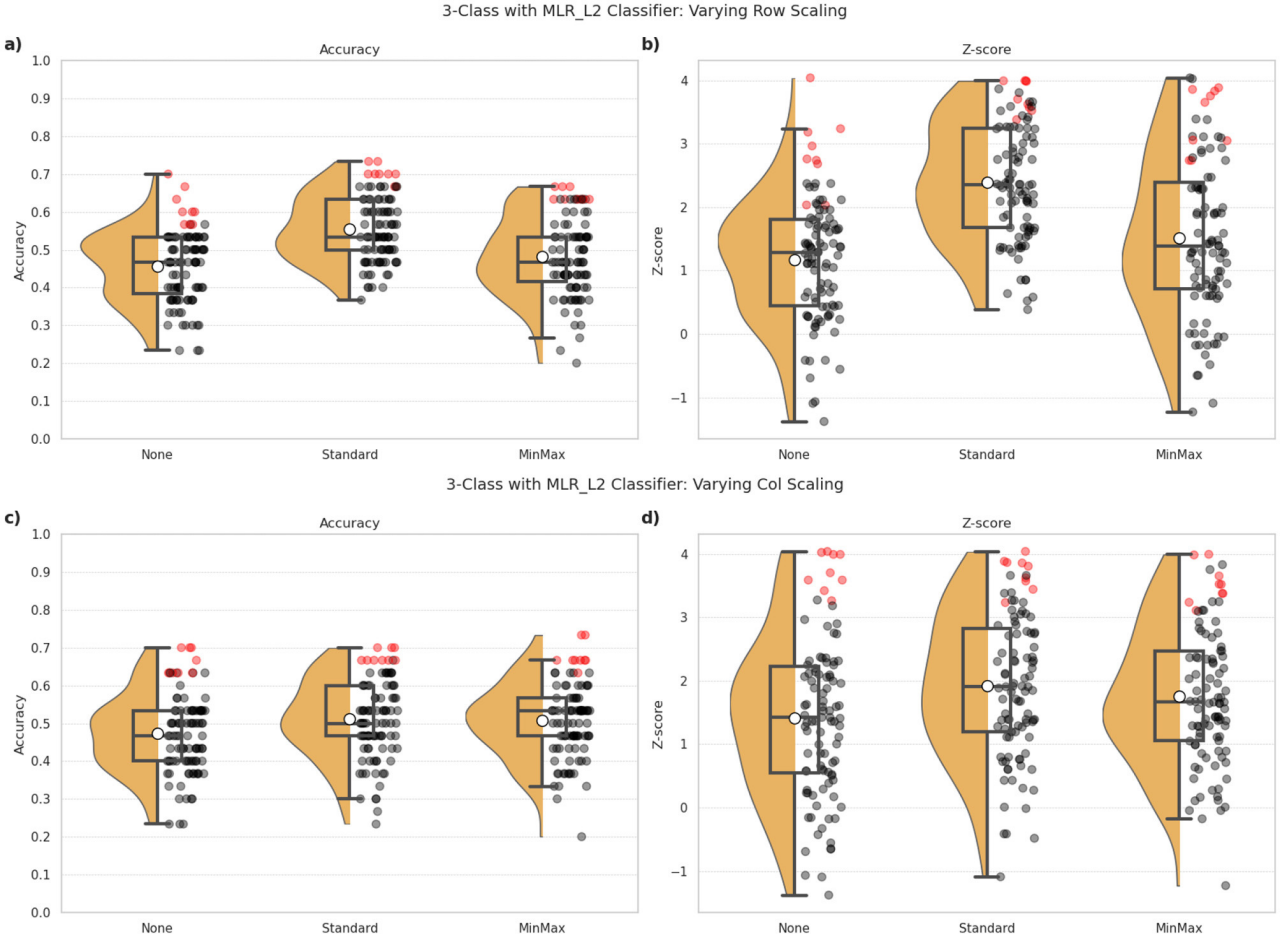
3-class classification performance observed for the MLR_L2 classifier under different combinations of row and column scaling approaches employed in the ML pipeline. **(a, c)** indicate the classification accuracy, and **(b, d)** show the Z-scores as indicators of robustness of the model's predictions. The top row **(a, b)** indicates the impact of different row (i.e., subject-wise) scaling approaches, namely: the absence of any scaling (None), StandardScaler and MinMaxScaler. The bottom row **(c, d)** indicates the impact of different column (i.e. feature-wise) scaling using the same techniques. The dots correspond to one for each combination of pipeline configuration evaluated for the classifier, i.e., 297 combinations per classifier in our study. Red dots highlight the top 10% of all configurations in terms of classification accuracy, and indicates the robustness of these predictions.

**Figure 3:**
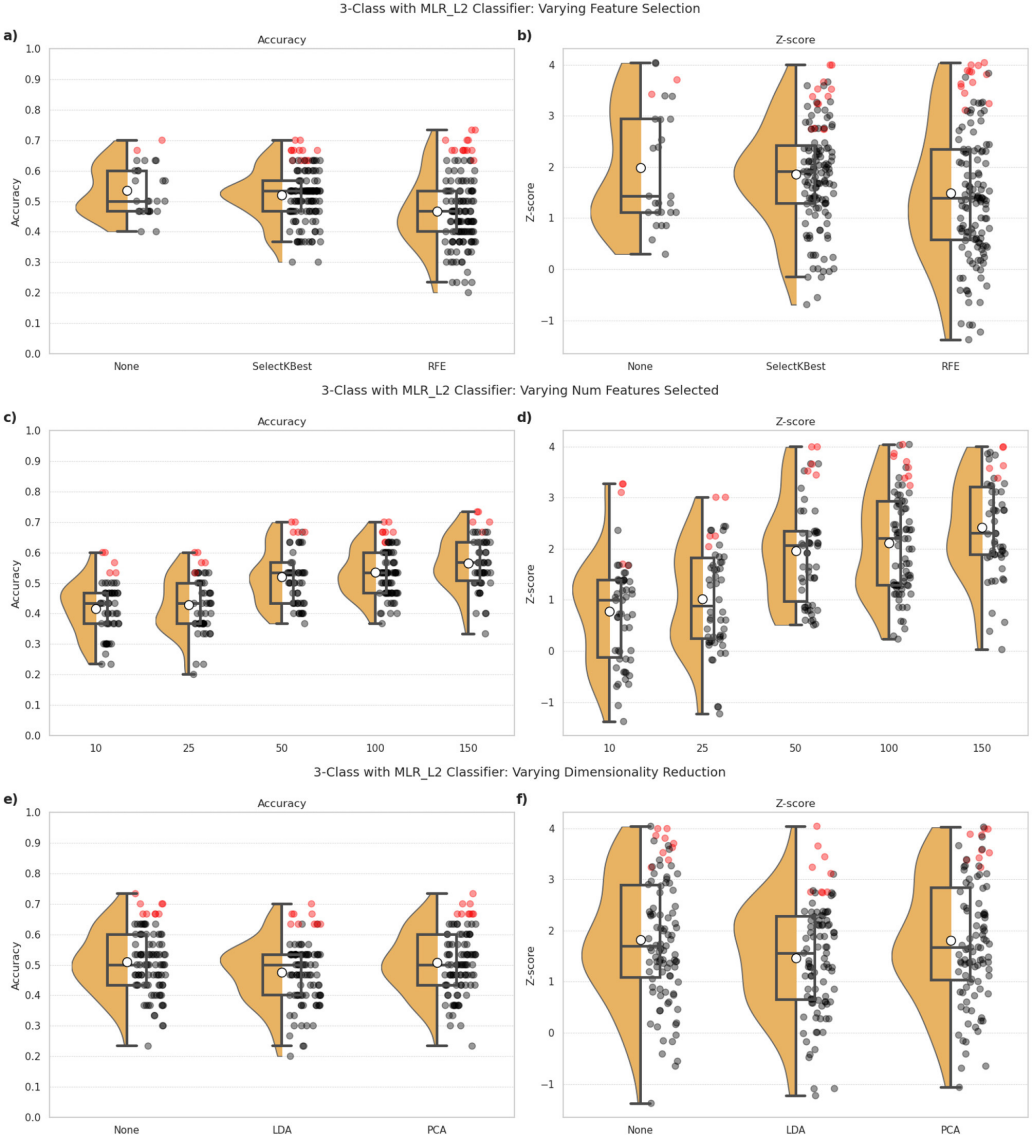
3-class classification performance observed for the MLR_L2 classifier under different combinations of feature selection and dimensionality reduction approaches employed in the ML pipeline. **(a, c, e)** indicate the classification accuracy, and **(b, d, f)** show the Z-scores as indicators of robustness of the model's predictions. The top row **(a, b)** indicates the impact of different feature selection approaches, namely: the absence of any feature selection (None), SelectKBest and RFE. The middle row **(c, d)** indicates the impact of different target feature counts when feature selection is part of the ML pipeline. The bottom row **(e, f)** indicates the impact of incorporating dimensionality reduction in the ML pipeline, via techniques such as LDA and PCA. The dots correspond to one for each combination of pipeline configuration evaluated for the classifier, i.e., 297 combinations per classifier in our study. Red dots highlight the top 10% of all configurations in terms of classification accuracy, and indicates the robustness of these predictions.

Figures 2, 3 illustrate the performance of the MLR_L2 classifier for 3-class classification under the various explored pipeline configurations. Through visualization of the distributions, variance, and top-performing configurations, the plots help highlight how the various pipeline design choices affect performance consistency. The inclusion of Z-scores further emphasizes model stability across different preprocessing approaches. Similar trends were demonstrated for the other classifiers as well. We have chosen to highlight the MLR_L2 classifier, as an example, in light of this model being a strong performer based on preliminary investigations.

#### 3.2.1 Impact of scaling methods

Figure 2 illustrates the performance of the MLR_L2 classifier under different scaling approaches. The top panels (a, b) assess the effect of row (subject-wise) scaling, while the bottom panels (c, d) focus on column (feature-wise) scaling. We evaluated three scaling methods: no scaling (None), StandardScaler, and MinMaxScaler.

##### 3.2.1.1 Row scaling

Classification accuracy (Figure 2a) showed certain notable differences across the three scaling methods, with StandardScaler generally performing better (mean accuracy = 0.55) than no scaling (mean accuracy = 0.46) and MinMaxScaler (mean accuracy = 0.48). However, these effects are more pronounced for the Z-scores (Figure 2b) with StandardScaler providing markedly more robust predictions (mean Z-score = 2.40), compared to the absence of scaling (mean Z-score = 1.78) and MinMaxScaler (mean Z-score = 1.52).

##### 3.2.1.2 Column scaling

The trends did not vary significantly between the different column scaling approaches (Figures 2c, d) with mean accuracies of 0.51, 0.50 and 0.47 for StandardScaler, MinMaxScaler, and (None) methods, respectively. In regard to robustness of the predictions, StandardScaler again show relatively superior outcomes (mean Z-score = 1.92), compared to MinMaxScaler (mean Z-score = 1.76) and no scaling (mean Z-score = 1.42). Notably, the absence of scaling (i.e., None) resulted in wider variability in outcomes.

**Figure 4:**
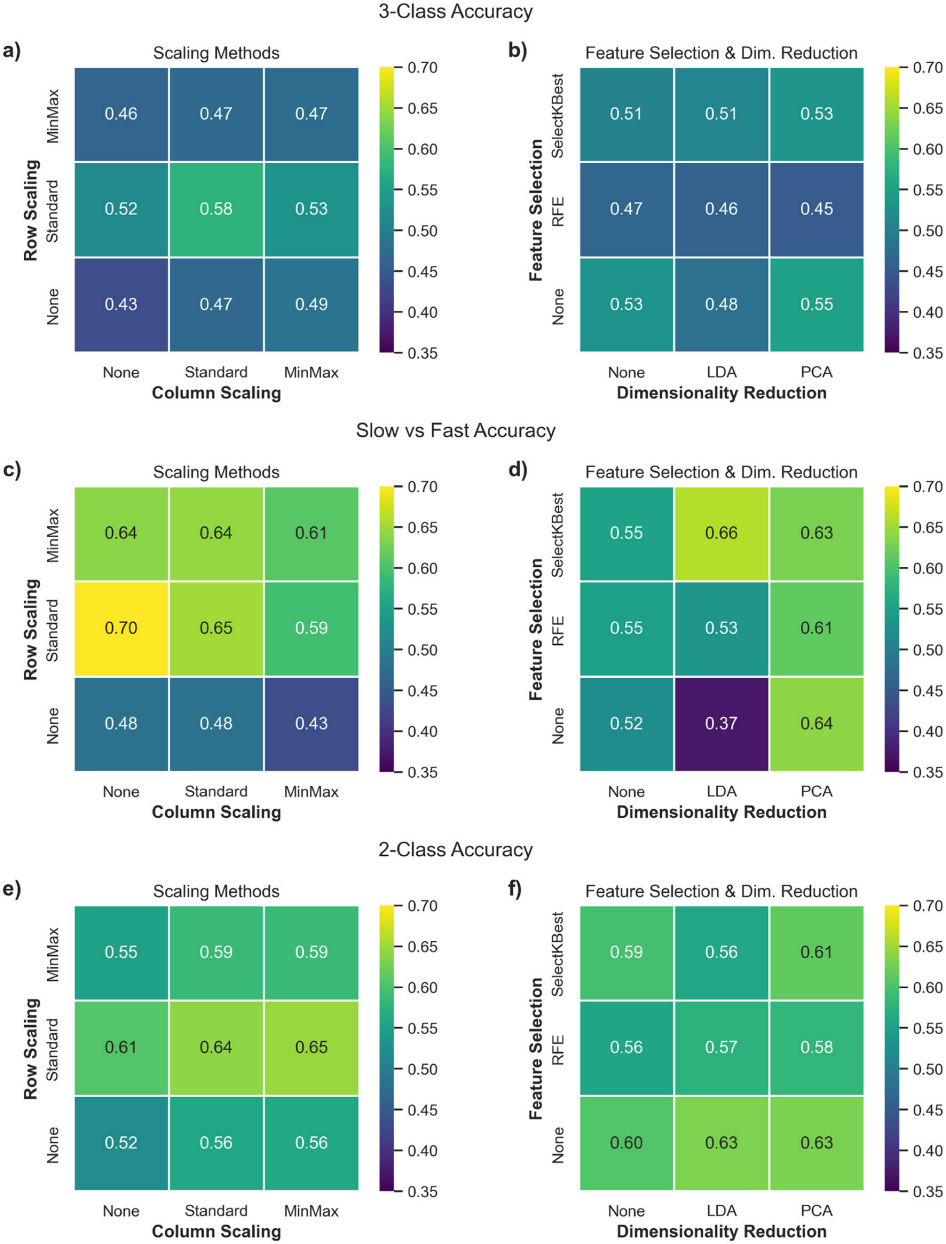
Heatmaps illustrating the average classification accuracy across various combinations of preprocessing components. Each cell represents the mean classification accuracy obtained across all 6 shortlisted classifiers for a given pipeline configuration. **(a, c, e)** compare row (subject-wise) vs. column (feature-wise) scaling approaches, while **(b, d, f)** compare feature selection methods vs. dimensionality reduction techniques. Panels correspond to the three classification tasks: **(a, b)**: 3-class (Controls vs. Slow ALS vs. Fast ALS), **(c, d)**: Slow vs. Fast ALS, **(e, f)**: 2-class (Controls vs. ALS).

Figures 4a, c, e, composed of data integrated across all classifiers, shows the effect of such scaling approaches across the three classification tasks. The most notable trend is that row-wise scaling—particularly using StandardScaler—consistently leads to higher classification accuracies across all three tasks. Overall, these results suggest that applying StandardScaler for both row and column scaling can help enhance the accuracy of predictions.

The Wilcoxon signed-rank test results for these configurations are presented in Table 1 when the MLR_L2 classifier was employed for the 3-class classification task. The analysis confirmed that the use of row-wise StandardScaler yielded consistent improvements in classification accuracy (*p* < 0.001; median: +10%). Column scaling also was found to be statistically significant, but with a much lower effect size (*p* < 0.001; median: +3.3%).

**Table 1:** Wilcoxon signed-rank test results for MLR_L2 classifier with the various pipeline steps and their different configurations.

**Pipeline step**	**Configuration**	***p*-value**	**Median %**	**Significance**	**n**
Row scaler	Standard	1.87e-13	+10.0%	***	99
Row scaler	MinMax	1.37e-02	+0.0%	*	99
Col scaler	MinMax	1.81e-04	+3.3%	***	99
Col scaler	Standard	3.60e-05	+3.3%	***	99
FeatSel-numfeats	RFE-10	2.23e-05	-16.7%	***	27
FeatSel-numfeats	RFE-25	1.17e-05	-13.3%	***	27
FeatSel-numfeats	RFE-50	3.66e-03	-6.7%	**	27
FeatSel-numfeats	RFE-100	6.87e-01	+0.0%		27
FeatSel-numfeats	RFE-150	3.13e-01	+3.3%		27
FeatSel-numfeats	SelectKBest-10	4.75e-05	-10.0%	***	27
FeatSel-numfeats	SelectKBest-25	3.80e-03	-6.7%	**	27
FeatSel-numfeats	SelectKBest-50	9.46e-02	+3.3%		27
FeatSel-numfeats	SelectKBest-100	9.44e-01	+0.0%		27
FeatSel-numfeats	SelectKBest-150	3.13e-02	+6.7%	*	27
Dim reduction	PCA	5.29e-02	+0.0%		99
Dim reduction	LDA	2.11e-04	-3.3%	***	99

Each configuration was compared against the absence of that step in the pipeline, for the 3-class classification task. The *p*-value indicates the level of significance while the Median % is used to indicate the effect size. *n* refers to the number of paired configurations; *: *p* < 0.05, **: *p* < 0.01, ***: *p* < 0.001.

#### 3.2.2 Influence of feature selection and dimensionality reduction

The impact of feature selection and dimensionality reduction on 3-class classification performance is detailed in Figure 3. Panels (a, b) explore different feature selection techniques, panels (c, d) vary the target feature count, and panels (e, f) evaluate dimensionality reduction approaches.

##### 3.2.2.1 Feature selection

As shown in Figures 3a, b, there didn't appear to be a significant improvement in the outcomes with the incorporation of feature selection techniques in the ML pipeline. The performance with SelectKBest (mean accuracy = 0.52) was comparable to that with no feature selection (mean accuracy = 0.53). RFE was found to perform most poorly for our classification task, in terms of both accuracy (mean accuracy = 0.47) and Z-scores.

##### 3.2.2.2 Number of features

Figures 3c, d illustrates the effect of various target feature counts, when feature selection is employed in the ML pipeline. The results reveal that selecting 50 features provided a good balance between accuracy and robustness, with increasing feature spaces providing only slight improvements in the outcomes.

##### 3.2.2.3 Dimensionality reduction

Figures 3e, f compares LDA and PCA with the absence of dimensionality reduction. Here again, we find that the classification outcomes did not benefit from the incorporation of this step in the ML pipeline. Both methods, LDA (mean accuracy = 0.48, mean Z-score = 1.47) and PCA (mean accuracy = 0.51, mean Z-score = 1.80), performed similar to pipelines without them (mean accuracy = 0.51, mean Z-score = 1.83). Dimensionality reduction, therefore, did not appear to have a notable improvement on the accuracy or robustness of the predictions, with LDA often yielding worse outcomes.

Figures 4b, d, f illustrate that the influence of feature selection and dimensionality reduction techniques varies across classification tasks and is generally limited in magnitude. In particular, RFE consistently underperforms compared to other configurations, suggesting that recursive elimination may discard informative features when applied to high-dimensional, small-sample datasets. Pipelines that exclude feature selection altogether or use simpler univariate methods such as SelectKBest tend to yield more stable results. Dimensionality reduction via PCA provides marginal improvements in some scenarios, though its contribution is often comparable to pipelines without any dimensionality reduction, indicating that extensive reduction of the feature space may not be necessary.

Table 1 lists the Wilcoxon signed-rank test results for these pipeline configurations. This shows with consistently high statistical signficance (*p* < 0.01), the unsuitability of performing feature selection on our dataset with a low number of target features (<50). Dimensionality reduction techniques, similarly, were found to yield no consistent benefit and in several cases significantly degraded performance. There is some improvement when a larger number of features are accommodated, suggesting the need of retaining sufficient feature dimensionality for the model to benefit from the rich, complementary information present in multimodal MRI data.

#### 3.2.3 Evaluating top performers

**Figure 5:**
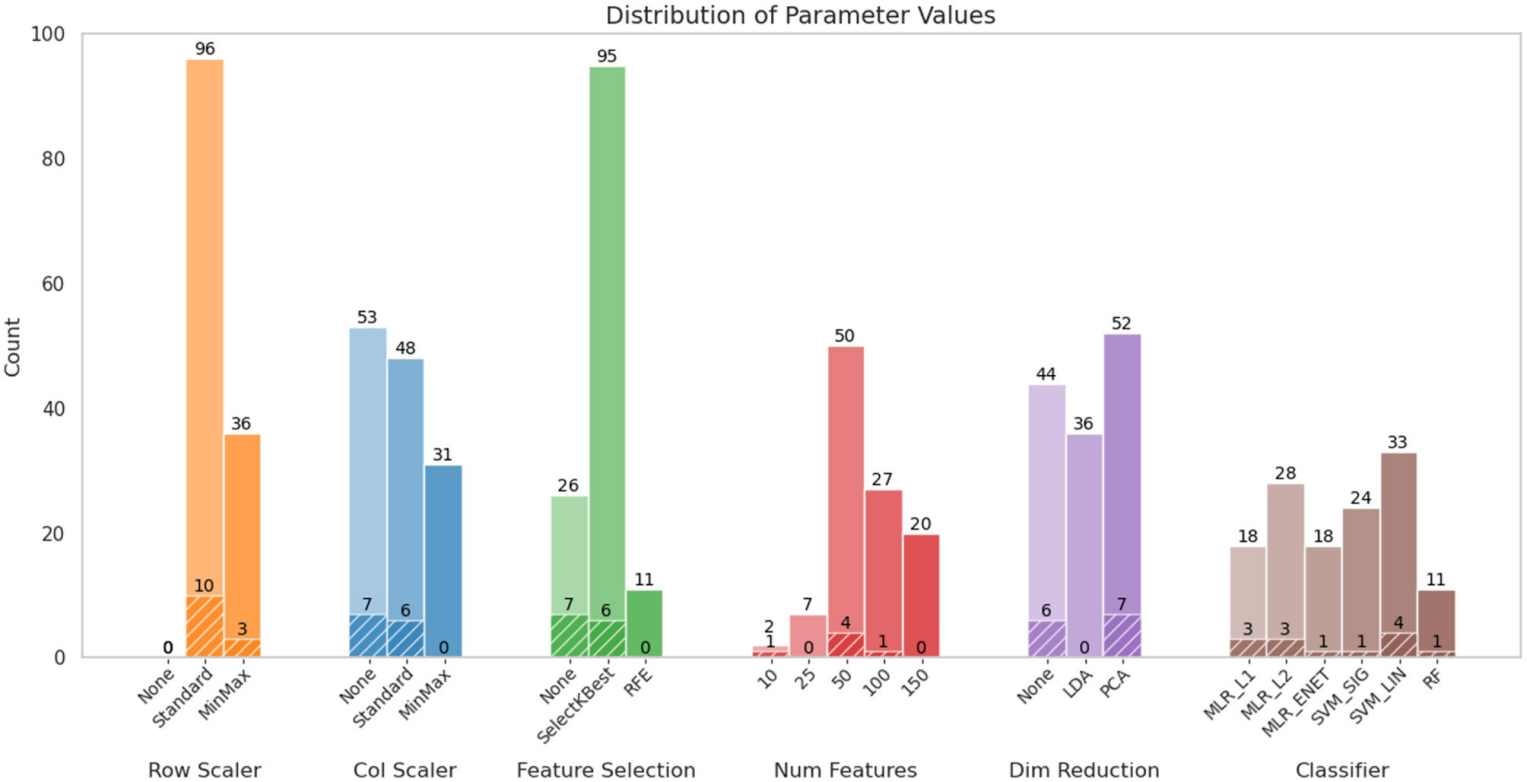
Distribution of various configurations of ML pipeline components. The colored bars represent those configurations where the 3-class and slow-vs.-fast accuracies ≥ 60%, along with their associated Z-scores ≥ 2.0. This corresponded to 132 out of the total of 1,782 combinations that were evaluated. The textured parts of the plots indicate the proportion of configurations where these classification accuracies were ≥ 70%; these accounted for only 13 combinations. Note that the parameter *Num Features* applies only when a feature selection method is involved (i.e., not None).

Figure 5 summarizes the distribution of parameter choices across pipeline configurations that achieved at least 60% classification accuracy for the 3-class and SvF tasks, with associated Z-scores of 2.0 or higher. It further highlights configurations that exhibited an even higher accuracy (≥ 70%). The key observations are: (i) StandardScaler emerged as a consistently effective choice for row scaling, (ii) column scaling appeared to have less influence on model performance, with MinMaxScaler notably absent among the top-performing configurations, (iii) there is a notable preponderance of SelectKBest as a feature selection method, often combined with 50 selected features, amongst the top performers. However, its prominence diminished when focusing on the highest-performing pipelines, where the absence of any feature selection stage proved to be equally effective, (iv) dimensionality reduction techniques did not demonstrate a clear benefit to the pipeline, with contribution of PCA to the best performers generally comparable to pipelines without any dimensionality reduction. By systematically evaluating these pipeline configurations, we identified the impact of the various steps and their configurations in optimizing classification outcomes, and also demonstrated the consistency of the findings with respect to specific choices for pipeline design.

### 3.3 Hyperparameter optimization

Based on the above findings, we proceeded with the design of the pipeline with a focus on optimizing the classifier hyperparameters. The pipeline employed StandardScaler to perform both row and column scaling. The feature selection and dimensionality reduction steps were excluded in view of their limited observed utility for our classification tasks. This allows us to isolate the impact of classifier settings on model performance, ensuring that we retain the most relevant features for the classification task without introducing unnecessary complexity.

**Figure 6:**
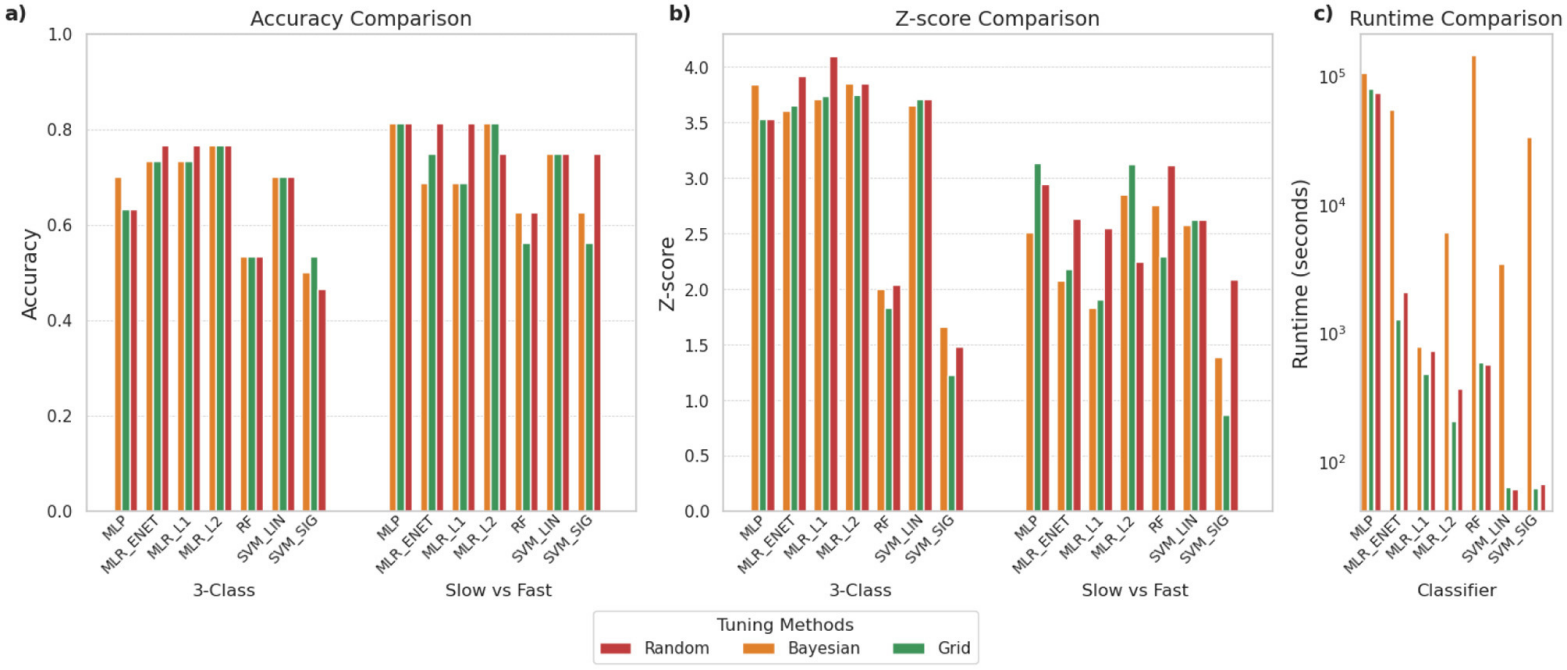
Comparison of different hyperparameter optimization approaches for the various shortlisted classifiers. The panels compare **(a)** classification accuracy, **(b)** Z-scores indicating robustness of predictions, and **(c)** time required for execution (in seconds), using log scale. To enable a fair comparison of the tuning methods for each classifier, the search spaces were standardized and the total number of parameter combinations tested was kept equal across all three methods.

As outlined earlier, we compared three different hyperparameter optimization approaches: (i) Grid Search, (ii) Random Search, and (iii) Bayesian Optimization. Figure 6 provides a comparison of the performance of these three approaches across the various classifiers we had shortlisted for both the 3-class and SvF classification tasks. It is observed that the classifiers exhibit a range of accuracies, but that these typically do not vary much based on the specific optimization strategy adopted in the pipeline, particularly for the 3-class classification task. Similarly, Z-score analysis does not point to a generally superior tuning procedure for achieving optimal outcomes. However, certain classifiers appear to benefit more from specific optimization strategies. For example, MLR_ENET was found to consistently yield better performances when optimized using the random search approach.

What is most notable is the differences in the execution time, with Bayesian optimization consistently requiring the longest time to complete. Grid search and random search were found to perform similarly in terms of computational efficiency. This pattern held across all classifier types.

### 3.4 Effect of dataset enrichment

Having explored various adjustments to the classification pipeline, we next examine potential benefits of dataset enrichment to compare the change in performance with respect to the fine-tuning of the ML pipeline. This can be achieved through several approaches, two of which are outlined below for evaluation. In view of their superior classification performance, as demonstrated in the previous sections, we decided to employ the MLR_L2 classifier with the grid search optimization strategy for the assessments ahead.

#### 3.4.1 Impact of multimodal data

Here, we examined the impact of integrating multiple MRI modalities on classification accuracy. Multimodal integration was tested to evaluate whether combining complementary sources of information—such as structural features from qT1, microstructural changes captured by DTI, and metabolic insights from ^23^Na imaging—could improve the model's ability to distinguish between subject groups. The rationale is that each modality provides unique and potentially non-redundant information about the underlying neuropathology, and their combination may enhance discriminative power (Cerasa et al., 2011).

**Figure 7:**
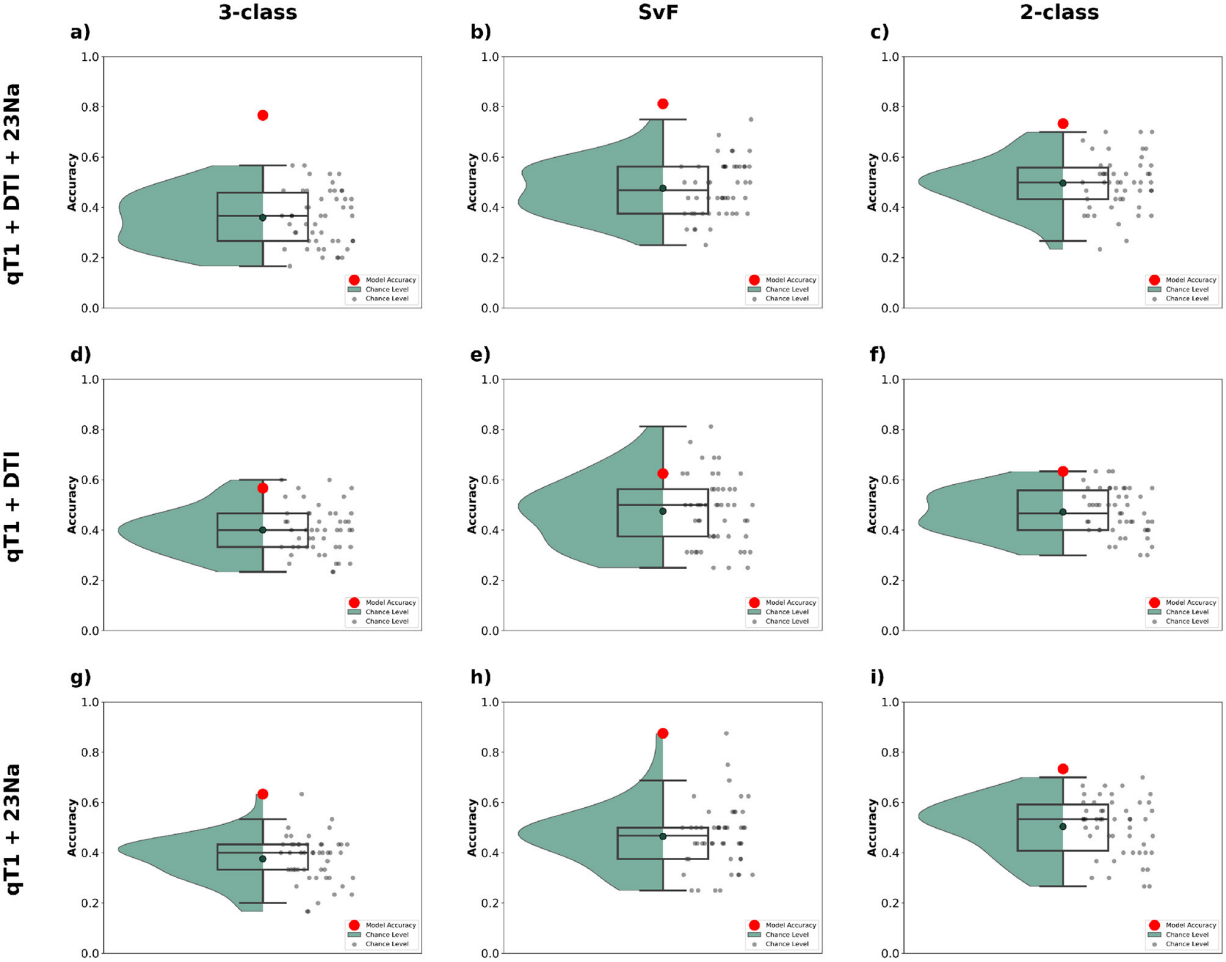
Classification performance observed using MLR_L2 classifier with different combinations of multimodal data. The top row **(a–c)** shows the outcomes when employing the complete dataset (qT1+DTI+^23^Na), the middle row **(d–f)** corresponds to the combination of qT1 and DTI modalities, and the bottom row **(g–i)** shows those for the combination of qT1 and ^23^Na modalities. The first column **(a, d, g)** show the classification accuracy for 3-class, the second column **(b, e, h)** for SvF, and the third column **(c, f, i)** for the 2-class task. The red markers indicate the classification accuracy of the model to the actual data. The black markers, and the green bounding region, indicate the accuracy of chance-level predictions observed via permutation testing.

The results are summarized in Figure 7, which compares performance across our three classification tasks: 3-class, SvF, and 2-class. It can be observed that combining all three modalities (i.e. qT1 + DTI + ^23^Na) yields the best classification performance across all tasks. The red markers representing model accuracy are consistently above the chance-level predictions, thereby indicating robustness of the model's predictions. The combination of qT1 + DTI is found to perform relatively poorly for all three tasks, while the combination of qT1 + ^23^Na shows more promise, with a notably high model accuracy for the SvF task. These findings underscore the utility of integrating multiple MRI modalities to improve classification accuracy.

#### 3.4.2 Impact of alternative parcellation strategies

ALS is known to involve both cortical and subcortical degeneration, with white matter alterations, particularly along corticospinal tracts and frontotemporal connections, emerging as key imaging biomarkers (Bede and Hardiman, 2018; Grapperon et al., 2019). A broader anatomical coverage, comprising of both the gray and white matter regions, could better reflect the underlying pathophysiology of ALS, and potentially help improve classification accuracy. We therefore investigated the impact of different brain parcellation strategies on classification accuracy for our tasks. Specifically, we compared results obtained using GM (gray matter) parcellation (containing 187 ROIs) to those using combined GM+WM (gray matter + white matter) parcellation (containing 332 ROIs).

**Figure 8:**
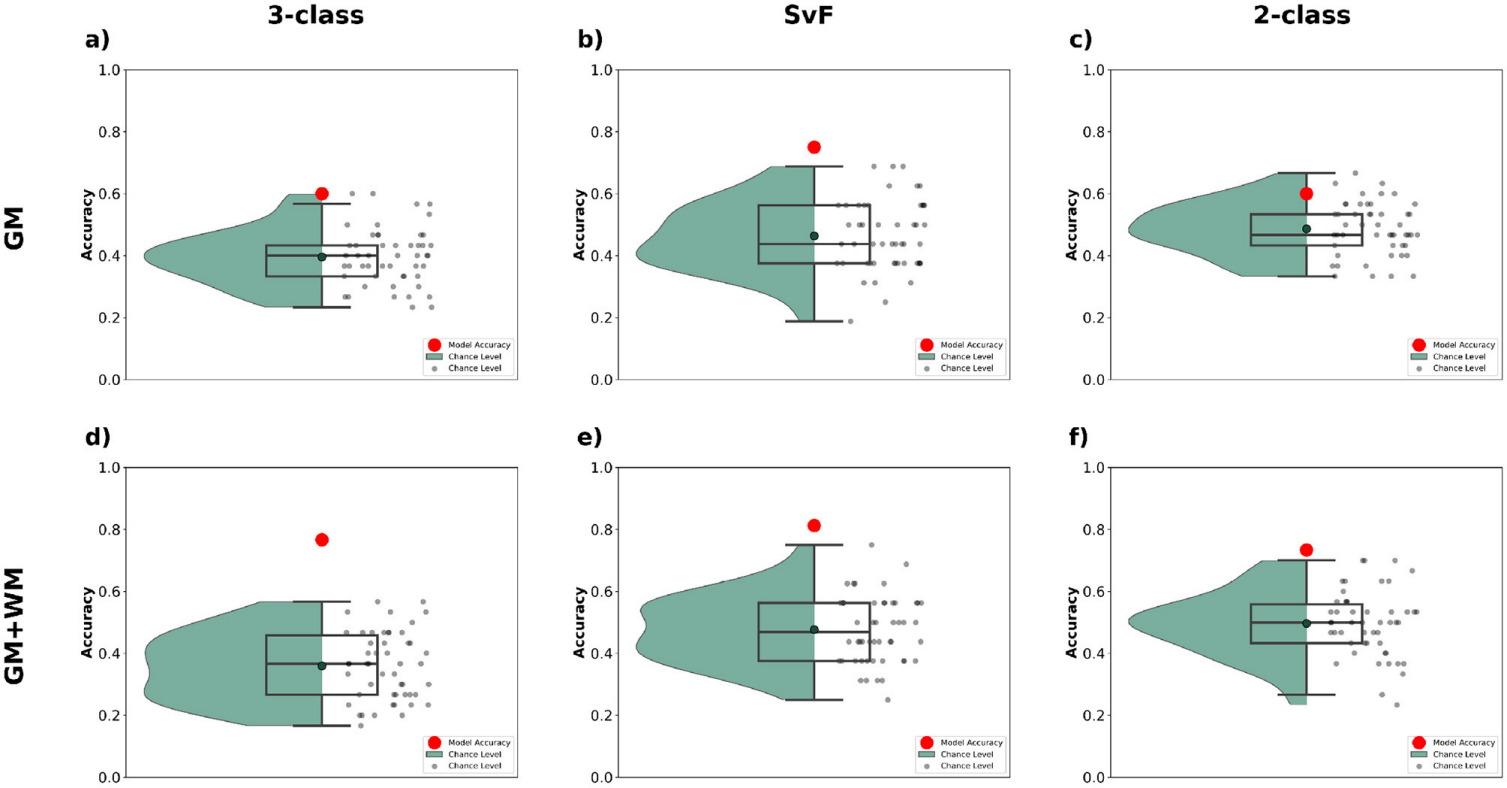
Classification performance observed via multimodal analysis using MLR_L2 classifier with different brain parcellations. The top row **(a–c)** shows the outcomes when employing the GM parcellation, and the bottom row **(d–f)** shows those for GM+WM parcellation. The first column **(a, d)** show the classification accuracy for 3-class, the second column **(b, e)** for SvF, and the third column **(c, f)** for the 2-class task. The red markers indicate the classification accuracy of the model to the actual data. The black markers, and the green bounding region, indicate the accuracy of chance-level predictions observed via permutation testing.

The results are presented in Figure 8 for the three classification tasks. We see that incorporating WM data alongside GM yields a notable improvement in classification accuracy across all tasks. For the 3-class task, the model achieves significantly higher accuracy with GM+WM, as compared to GM alone, indicating that the more comprehensive parcellation helps the model capture additional discriminative features crucial for distinguishing between the classes. Similarly, for the SvF and 2-class tasks, the inclusion of WM data results in a marked boost in performance, with the model accuracy consistently positioned higher than chance-level predictions.

## 4 Discussion

This study systematically evaluated the design and performance of machine learning pipelines for small-cohort multimodal neuroimaging data, using ALS as a case study. By focusing on the effects of pipeline configurations, such as scaling, feature selection, dimensionality reduction, and hyperparameter optimization, we aimed to understand the extent to which these elements impact classification outcomes, and compare their influence to the enrichment of the datasets. Our findings highlight several key insights, while also underscoring the limitations of such small-cohort studies and the importance of dataset enrichment.

The primary conclusion of this study is that the choice of ML pipeline configurations had only modest effects on classification performance. Despite evaluating a comprehensive range of approaches, the differences in performance were limited. We observed that row scaling (subject-wise) using StandardScaler consistently improved classification outcomes, while column scaling (feature-wise) had less noticeable impact. This suggests that, when measurements vary across subjects, the critical information may lie in the relative ranking of values—such as identifying regions with the highest or lowest intensities, in line with previous findings on within-session normalization for effective connectivity estimated from fMRI data for decoding (Pallarés et al., 2018; Gravel et al., 2020). Feature selection and dimensionality reduction steps did not significantly enhance performance. In many cases, omitting these steps altogether produced results comparable to, or better than, their inclusion in the ML pipeline. Hyperparameter optimization provided some performance gains, but the choice of optimization strategy (grid search, random search, or Bayesian optimization) typically had limited impact on the overall accuracy. Based on our results, the choice of hyperparameter tuning strategy should consider the trade-offs between accuracy, robustness, and computational efficiency. For instance, Bayesian Optimization may be more suitable for scenarios where achieving optimal performance is critical and computational resources are not a constraint, whereas Random Search or Grid Search may suffice for applications with tighter time or resource limitations.

To design an efficient machine learning workflow, it is advisable to start by evaluating classifiers in their default configurations to establish a performance baseline. Data preprocessing techniques should be systematically evaluated (e.g., scaling, feature selection, dimensionality reduction) to determine their effectiveness for the given dataset. Once the different pipeline components are selected, various hyperparameters can be optimized to assess generalizability. Such a staged approach to pipeline design enables informed decisions aimed at performance improvements while minimizing the computational costs.

While pipeline refinement is essential for maximizing performance, its impact is inherently constrained when the dataset is small and limited in its ability to generalize. This suggests that, for small-cohort studies, significant efforts devoted to refining ML pipelines may not yield proportional improvements in outcomes. Unconventional approaches to deal with limited patient datasets may also enable us to readily improve the model performance, such as hybrid model training wherein larger control groups have been employed in the training phase, but combined with another class (e.g. slow ALS) in the validation phase for stratification of patients based on disease progression (e.g. slow vs. fast ALS). This approach has been found effective in an ongoing study involving the same datasets employed here, wherein this hybrid approach enabled us to attain a classification accuracy of 88% for the SvF task. It should be borne in mind that class labels can frequently involve a degree of uncertainty in clinical studies, and therefore classification studies often do not yield very high accuracies. For instance, the slow and fast progressors were evaluated here based on a single cross-sectional ALSFRS-R slope. However, ALS progression is heterogeneous, and such snapshot metrics may not capture the full clinical trajectory. While longitudinal imaging could help address this limitation to some extent, it is often challenging to obtain this for ALS studies, leaving us reliant on cross-sectional data.

Greater emphasis might be placed on addressing the limitations inherent to the dataset itself. There are several approaches to tackling this problem. The emergence of ultra-high-field MRI systems, such as 7T MRI, has dramatically improved spatial and temporal resolution, enabling the detection of microstructural and metabolic changes with unparalleled precision. For example, studies have reported that 7T MRI enhances the contrast and visibility of brain tumors compared to those acquired at 3T (Noebauer-Huhmann et al., 2015). This suggests that, even with limited data cohorts, scans obtained at 7T might be more informative than those obtained at 3T and 1.5T. The scope of multimodal neuroimaging can be extended by combining neuroimaging data with other data types, such as clinical, genetic, and biochemical data. These additional data sources can provide complementary insights into disease mechanisms that are not evident from imaging data alone (Menke et al., 2017; Iturria-Medina et al., 2021). Efforts should also be made at progressively increasing the size of the cohorts wherever possible. Data augmentation is often a useful step in expanding datasets, especially in scenarios where acquiring additional samples is challenging. There exists a variety of techniques by which this can be achieved, such as via geometric/intensity transformations or synthetic data generation (Garcea et al., 2023; Chintapalli et al., 2024), such as generative models (e.g., GANs, VAEs), to simulate realistic variability and enhance representation of under-sampled phenotypes (Chadebec et al., 2022; Kebaili et al., 2023). Multi-centric approaches offer another option for pooling together datasets to obtain larger cohorts, though this is often accompanied by significant data harmonization issues (Stamoulou et al., 2022).

While subsets of modalities can provide improved performance, as compared to single modality outcomes, the combined use of all available modalities helps capture complementary features and potential synergistic effects that enhance model performance. This was evident in our results, wherein the best outcomes were observed under multimodal analysis of all modalities. This highlights the importance of acquiring and merging as many relevant modalities as possible for comprehensive analysis, thereby helping orient the data collection process. Further, our findings regarding variations in the parcellation strategy highlights the importance of adopting techniques to maximize the information extracted from already available datasets. While GM parcellation alone provides meaningful insights, the addition of WM data enhances the model's ability to differentiate between classes. These underscore the value of employing more comprehensive methods in clinical analyses to improve classification outcomes and better exploit the full potential of existing datasets (Kobeleva et al., 2022). The above findings, taken together, emphasize the critical role of dataset quality over ML pipeline complexity in small-cohort studies.

Our findings concur with the conclusions drawn by Dadi et al. (2019) who systematically benchmarked machine learning pipelines for resting-state functional MRI (fMRI) on larger datasets. Similarly to our study, they highlighted the sensitivity of prediction outcomes to various choices in the pipeline design. Both studies underscore that careful optimization of preprocessing and analytical steps is essential, particularly when dealing with small or heterogeneous datasets. Both studies stress the importance of computational simplicity and robustness. For instance, Dadi et al. (2019) demonstrated that linear models (e.g., logistic regression) performed consistently well across datasets; our study similarly found these group of classifiers effective and also that simpler pipelines often yield comparable performance to more complex configurations. This consistency suggests that general trends in pipeline optimization can apply across neuroimaging modalities.

While classification accuracy alone does not determine clinical usability, our findings offer preliminary insights into how imaging-based biomarkers can contribute to future diagnostic or prognostic tools in ALS. It is important to emphasize that MRI is not currently a standard tool for ALS diagnosis, which still relies heavily on clinical examination and electrophysiological assessments. However, as imaging techniques and ultra-high-field MRI become more effective in capturing microstructural and metabolic alterations, there is growing interest in incorporating MRI-derived biomarkers to complement traditional techniques. In this context, our study provides a methodological foundation by systematically evaluating the robustness of different ML pipeline components under realistic, small-cohort conditions. In this light, a 70%–80% accuracy in distinguishing among controls, slow, and fast ALS patients is not proposed as a definitive diagnostic tool, but rather as a proof-of-concept suggesting that quantitative multimodal MRI contains clinically relevant information about disease state and progression rate.

Another critical factor for the clinical integration of ML models is related to model interpretability. While the primary focus of our study was on benchmarking pipeline configurations, it is worth noting that several classifiers we employed (e.g., MLR_L2, MLR_L1, MLR_ENET, SVM_LIN) offer inherent interpretability through feature weight analysis. Interpretability methods are best used in conjunction with classifiers that exhibit a sufficiently good level of accuracy, and our focus here has been on evaluating pipeline design to enable this. In a future study, we intend to extend this work by incorporating *post hoc* explainability methods, such as SHAP or LIME (Thibeau-Sutre et al., 2023), to further elucidate the biological relevance of the identified imaging-based biomarkers, and to identify which brain regions or modalities drive classification decisions. Enhancements to the models presented here, such as through implementation of intrepretbility of model outcomes, could assist neurologists in prioritizing follow-up assessments, identifying atypical progression patterns, or selecting patients for trials. Also, even moderate accuracies can have value when used as a decision support tool rather than a diagnostic replacement.

While our study focuses specifically on ALS, the challenges and methodological considerations discussed here are broadly shared across many rare neurological conditions, including Huntington's disease (Andica et al., 2020), Multiple System Atrophy (Wan et al., 2023) and Creutzfeldt-Jakob Disease (Baiardi et al., 2023). In such contexts, patient recruitment, imaging harmonization, and phenotypic heterogeneity often result in small, heterogeneous datasets. The general conclusion that pipeline refinement yields modest gains when data are limited, and that dataset enrichment or multimodal integration has greater impact, is expected to extend beyond ALS. Conversely, some effects observed here—such as minimal improvements from model tuning—may diminish as dataset sizes increase and model generalization improves.

In conclusion, while optimizing the ML pipeline remains an important aspect of AI-driven neuroimaging research, our findings indicate that, in small-cohort studies, extensive pipeline tuning may often yield only modest improvements in performance. Efforts should also focus on dataset enrichment through larger cohorts, multimodal data integration, and improved preprocessing techniques. These steps are likely to have a more substantial impact on advancing the clinical utility of ML-based approaches in neuroimaging. This work may serve as a reference for researchers developing ML frameworks in similarly constrained domains, while highlighting the need for larger, more diverse datasets and integrative modeling approaches.

## Data Availability

The raw data that supports the findings of this study are available from the authors upon reasonable request.
