# Supplementary material for: Evaluating machine learning pipelines for multimodal neuroimaging in small cohorts: an ALS case study
Source: Front Neuroinform. 2025 Jun 13;19:1568116. doi: 10.3389/fninf.2025.1568116 (PMC12202540; doi:10.3389/fninf.2025.1568116)
Supplement: Supplementary file 1 [file Data_Sheet_1.pdf]

Evaluating Machine Learning Pipelines for Multimodal Neuroimaging in Small Cohorts: An ALS Case Study

- *Supplementary Material*

1 ML PIPELINE FLOWCHART

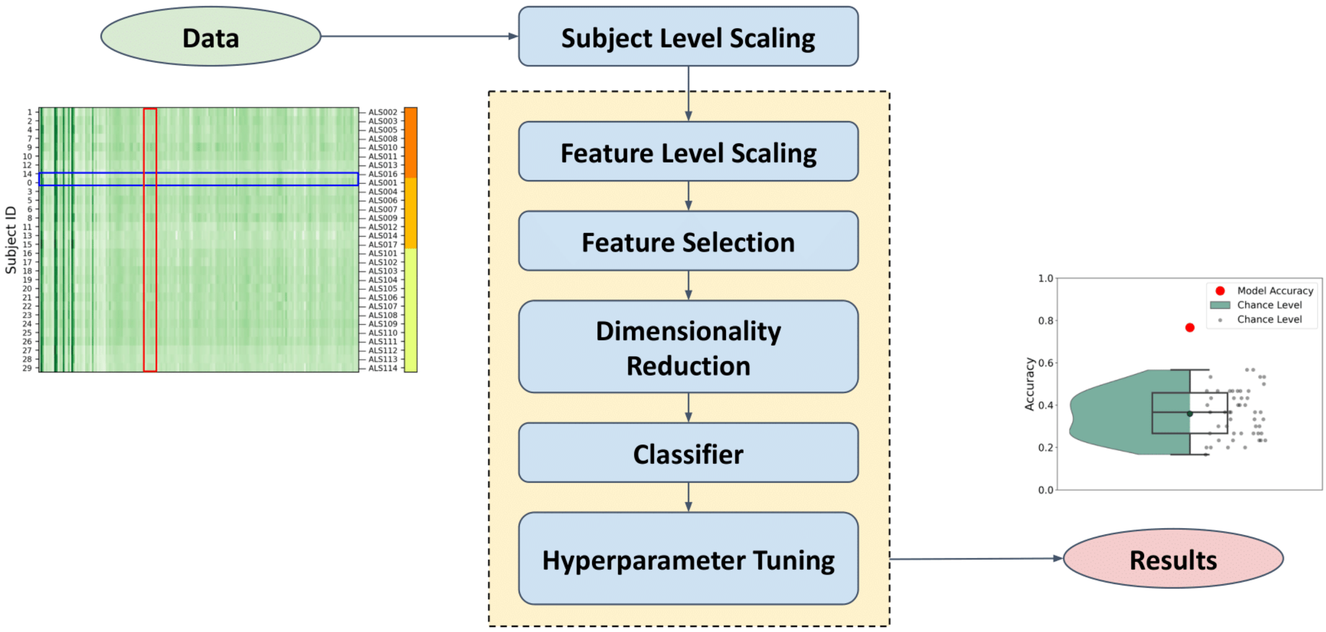

Figure S1: A schematic representation of the classification pipeline used in this study. Raw multimodal imaging data (matrix on the left; rows = subjects, columns = features) undergoes an initial subject-level scaling (blue box in matrix) step to account for inter-subject variability. The core processing pipeline (highlighted yellow box) consists of feature-level scaling (red box in matrix), optional feature selection (e.g., SelectKBest, RFE), and/or dimensionality reduction (e.g., PCA, LDA), followed by classification using one of the selected classifiers. Model hyperparameters are optimized through inner-loop cross-validation. Performance metrics (right) are evaluated on held-out test samples using LOOCV, and results are compared against chance levels via permutation testing.

2 HYPERPARAMETER OPTIMIZATION: SEARCH SPACES

Table S1: Hyperparameter search spaces for various classifiers. The search spaces were standardized, and the total number of tested parameter combinations was kept consistent across all three methods.

Model	Grid Search	Random Search	Bayesian Optimization
**Random Forest (RF)**			
n_{trees}	$\{50, 150, 300\}$	$[50, 300]$, step 25	$[50, 300]$
max_depth	$\{-, 10, 20\}$	$\{-, [5, 20]\}$, step 5	$[5, 20]$
min_samples_split	$\{2, 5, 10\}$	$\{2, 5, 10\}$	$[2, 10]$
min_samples_leaf	$\{1, 2, 4\}$	$\{1, 2, 4\}$	$[1, 4]$
max_features	{sqrt, log2}	{sqrt, log2}	{sqrt, log2}
**Logistic Regression - L1 (MLR_L1)**			
C	$\{0.01, 0.1, 1, 10, 100\}$	$U(0.01, 100)$	$\log U(0.01, 100)$
solver	saga	saga	saga
max_iter	$\{100, 300, 500, 1000\}$	$[100, 1000]$	$[100, 1000]$
**Logistic Regression - L2 (MLR_L2)**			
C	$\{0.01, 0.1, 1, 10, 100\}$	$U(0.01, 100)$	$\log U(0.01, 100)$
solver	{lbfgs, saga}	{lbfgs, saga}	{lbfgs, saga}
max_iter	$\{100, 300, 500, 1000\}$	$[100, 1000]$	$[100, 1000]$
**Logistic Regression - Elastic Net (MLR_ENET)**			
C	$\{0.01, 0.1, 1, 10, 100\}$	$U(0.01, 100)$	$\log U(0.01, 100)$
$l1$_ratio	$\{0.1, 0.3, 0.5, 0.7, 0.9\}$	$U(0.1, 0.9)$	$[0.1, 0.9]$
max_iter	$\{100, 300, 500, 1000\}$	$[100, 1000]$	$[100, 1000]$
**SVM - Linear (SVM_LIN)**			
C	$\{0.01, 0.1, 1, 10, 100\}$	$U(0.01, 100)$	$\log U(0.01, 100)$
max_iter	$\{100, 500, 1000\}$	$[100, 1000]$	$[100, 1000]$
tol	$\{10^{-4}, 10^{-3}, 10^{-2}\}$	$\log U(10^{-4}, 10^{-2})$	$\log U(10^{-4}, 10^{-2})$
**SVM - Sigmoid (SVM_SIG)**			
C	$\{0.01, 0.1, 1, 10, 100\}$	$U(0.01, 100)$	$\log U(0.01, 100)$
γ	$\{0.001, 0.01, 0.1, 1\}$	$U(0.001, 1)$	$\log U(0.001, 1)$
coef0	$\{0, 0.5, 1, 2\}$	$U(0, 2)$	$[0, 2]$
**Multi-Layer Perceptron (MLP)**			
hidden_layers	$\{(50), (100), (50, 50)\}$	$\{(50), (100), (50, 50)\}$	$\{(50), (100), (50, 50)\}$
activation	{relu, tanh}	{relu, tanh}	{relu, tanh}
α	$\{10^{-4}, 10^{-3}, 10^{-2}\}$	$U(10^{-4}, 10^{-2})$	$\log U(10^{-4}, 10^{-2})$
learning_rate	{constant, invscaling}	{constant, invscaling}	{constant, invscaling}
max_iter	$\{200, 600, 1000\}$	$[200, 1000]$	$[200, 1000]$

3 WILCOXON SIGNED-RANK TEST: ALL CLASSIFIERS

Table S2: Wilcoxon signed-rank test results for all the classifiers with the various pipeline steps and their different configurations. Each configuration was compared against the absence of that step in the pipeline, for the 3-class classification task. The p-value indicates the level of significance while the Median % is used to indicate the effect size. Note: n refers to the number of paired configurations; *: $p < 0.05$, **: $p < 0.01$, ***: $p < 0.001$

Pipeline Step	Configuration	Classifier	p-value	Median %	Significance	n
Row Scaler	Standard	MLR_L1	4.76e-06	+6.7%	***	99
Row Scaler	MinMax	MLR_L1	2.92e-01	+0.0%		99
Row Scaler	Standard	MLR_ENET	1.10e-10	+10.0%	***	99
Row Scaler	MinMax	MLR_ENET	9.22e-01	+0.0%		99
Row Scaler	Standard	SVM_LIN	1.96e-10	+6.7%	***	99
Row Scaler	MinMax	SVM_LIN	2.93e-03	+0.0%	**	99
Row Scaler	Standard	MLR_L2	1.87e-13	+10.0%	***	99
Row Scaler	MinMax	MLR_L2	1.37e-02	+0.0%	*	99
Row Scaler	Standard	SVM_SIG	4.82e-09	+6.7%	***	99
Row Scaler	MinMax	SVM_SIG	2.07e-01	+0.0%		99
Row Scaler	Standard	RF	1.39e-11	+6.7%	***	99
Row Scaler	MinMax	RF	1.07e-04	-6.7%	***	99
Col Scaler	Standard	MLR_L1	3.47e-05	+3.3%	***	99
Col Scaler	MinMax	MLR_L1	2.19e-03	+0.0%	**	99
Col Scaler	Standard	MLR_ENET	7.03e-07	+3.3%	***	99
Col Scaler	MinMax	MLR_ENET	4.91e-08	+3.3%	***	99
Col Scaler	MinMax	SVM_LIN	3.65e-02	+0.0%	*	99
Col Scaler	Standard	SVM_LIN	1.00e-01	+0.0%		99
Col Scaler	MinMax	MLR_L2	1.81e-04	+3.3%	***	99
Col Scaler	Standard	MLR_L2	3.60e-05	+3.3%	***	99
Col Scaler	Standard	SVM_SIG	1.59e-02	+0.0%	*	99
Col Scaler	MinMax	SVM_SIG	3.29e-01	+0.0%		99
Col Scaler	Standard	RF	1.80e-01	+0.0%		99
Col Scaler	MinMax	RF	8.55e-02	+0.0%		99
FeatSel-NumFeats	RFE-10	MLR_L1	4.86e-05	-13.3%	***	27
FeatSel-NumFeats	RFE-25	MLR_L1	6.40e-04	-13.3%	***	27
FeatSel-NumFeats	RFE-50	MLR_L1	1.31e-02	-6.7%	*	27
FeatSel-NumFeats	RFE-100	MLR_L1	4.34e-01	-3.3%		27
FeatSel-NumFeats	RFE-150	MLR_L1	2.74e-01	-3.3%		27
FeatSel-NumFeats	SelectKBest-10	MLR_L1	5.56e-05	-10.0%	***	27
FeatSel-NumFeats	SelectKBest-25	MLR_L1	3.98e-04	-6.7%	***	27
FeatSel-NumFeats	SelectKBest-50	MLR_L1	3.58e-01	+0.0%		27
FeatSel-NumFeats	SelectKBest-100	MLR_L1	3.12e-01	+0.0%		27
FeatSel-NumFeats	SelectKBest-150	MLR_L1	3.09e-01	+3.3%		27

continued...

Pipeline Step	Configuration	Classifier	p-value	Median %	Significance	n
FeatSel-NumFeats	RFE-10	MLR_ENET	2.54e-05	-13.3%	***	27
FeatSel-NumFeats	RFE-25	MLR_ENET	3.25e-04	-10.0%	***	27
FeatSel-NumFeats	RFE-50	MLR_ENET	6.12e-03	-6.7%	**	27
FeatSel-NumFeats	RFE-100	MLR_ENET	4.64e-01	-3.3%		27
FeatSel-NumFeats	RFE-150	MLR_ENET	6.67e-01	+0.0%		27
FeatSel-NumFeats	SelectKBest-10	MLR_ENET	6.66e-06	-10.0%	***	27
FeatSel-NumFeats	SelectKBest-25	MLR_ENET	1.18e-03	-6.7%	**	27
FeatSel-NumFeats	SelectKBest-50	MLR_ENET	4.26e-01	+0.0%		27
FeatSel-NumFeats	SelectKBest-100	MLR_ENET	2.24e-01	+3.3%		27
FeatSel-NumFeats	SelectKBest-150	MLR_ENET	3.63e-01	+3.3%		27
FeatSel-NumFeats	RFE-10	SVM_LIN	2.09e-07	-16.7%	***	27
FeatSel-NumFeats	RFE-25	SVM_LIN	7.93e-06	-16.7%	***	27
FeatSel-NumFeats	RFE-50	SVM_LIN	1.25e-03	-16.7%	**	27
FeatSel-NumFeats	RFE-100	SVM_LIN	4.61e-03	-13.3%	**	27
FeatSel-NumFeats	RFE-150	SVM_LIN	4.11e-02	-3.3%	*	27
FeatSel-NumFeats	SelectKBest-10	SVM_LIN	3.94e-04	-10.0%	***	27
FeatSel-NumFeats	SelectKBest-25	SVM_LIN	2.49e-03	-6.7%	**	27
FeatSel-NumFeats	SelectKBest-50	SVM_LIN	6.78e-02	+0.0%		27
FeatSel-NumFeats	SelectKBest-100	SVM_LIN	2.89e-01	+0.0%		27
FeatSel-NumFeats	SelectKBest-150	SVM_LIN	2.11e-01	+3.3%		27
FeatSel-NumFeats	RFE-10	MLR_L2	2.23e-05	-16.7%	***	27
FeatSel-NumFeats	RFE-25	MLR_L2	1.17e-05	-13.3%	***	27
FeatSel-NumFeats	RFE-50	MLR_L2	3.66e-03	-6.7%	**	27
FeatSel-NumFeats	RFE-100	MLR_L2	6.87e-01	+0.0%		27
FeatSel-NumFeats	RFE-150	MLR_L2	3.13e-01	+3.3%		27
FeatSel-NumFeats	SelectKBest-10	MLR_L2	4.75e-05	-10.0%	***	27
FeatSel-NumFeats	SelectKBest-25	MLR_L2	3.80e-03	-6.7%	**	27
FeatSel-NumFeats	SelectKBest-50	MLR_L2	9.46e-02	+3.3%		27
FeatSel-NumFeats	SelectKBest-100	MLR_L2	9.44e-01	+0.0%		27
FeatSel-NumFeats	SelectKBest-150	MLR_L2	3.13e-02	+6.7%	*	27
FeatSel-NumFeats	RFE-10	SVM_SIG	1.60e-04	-10.0%	***	27
FeatSel-NumFeats	RFE-25	SVM_SIG	3.52e-04	-13.3%	***	27
FeatSel-NumFeats	RFE-50	SVM_SIG	3.84e-03	-6.7%	**	27
FeatSel-NumFeats	RFE-100	SVM_SIG	5.08e-01	+0.0%		27
FeatSel-NumFeats	RFE-150	SVM_SIG	9.50e-02	+0.0%		27
FeatSel-NumFeats	SelectKBest-10	SVM_SIG	2.90e-02	+0.0%	*	27
FeatSel-NumFeats	SelectKBest-25	SVM_SIG	1.41e-01	+0.0%		27
FeatSel-NumFeats	SelectKBest-50	SVM_SIG	1.79e-02	+3.3%	*	27
FeatSel-NumFeats	SelectKBest-100	SVM_SIG	2.26e-04	+6.7%	***	27
FeatSel-NumFeats	SelectKBest-150	SVM_SIG	4.00e-04	+6.7%	***	27
FeatSel-NumFeats	RFE-10	RF	5.31e-04	-10.0%	***	27

continued...

Pipeline Step	Configuration	Classifier	p-value	Median %	Significance	n
FeatSel-NumFeats	RFE-25	RF	4.39e-02	-6.7%	*	27
FeatSel-NumFeats	RFE-50	RF	8.51e-01	-3.3%		27
FeatSel-NumFeats	RFE-100	RF	2.74e-01	+3.3%		27
FeatSel-NumFeats	RFE-150	RF	4.81e-01	+3.3%		27
FeatSel-NumFeats	SelectKBest-10	RF	5.64e-02	+0.0%		27
FeatSel-NumFeats	SelectKBest-25	RF	2.97e-01	-3.3%		27
FeatSel-NumFeats	SelectKBest-50	RF	1.97e-01	+3.3%		27
FeatSel-NumFeats	SelectKBest-100	RF	2.58e-01	+3.3%		27
FeatSel-NumFeats	SelectKBest-150	RF	1.19e-01	+3.3%		27
Dim Reduction	PCA	MLR_L1	7.02e-01	+0.0%		99
Dim Reduction	LDA	MLR_L1	7.40e-01	+0.0%		99
Dim Reduction	PCA	MLR_ENET	2.50e-01	+0.0%		99
Dim Reduction	LDA	MLR_ENET	6.57e-02	-3.3%		99
Dim Reduction	LDA	SVM_LIN	7.57e-02	+0.0%		99
Dim Reduction	PCA	MLR_L2	5.29e-02	+0.0%		99
Dim Reduction	LDA	MLR_L2	2.11e-04	-3.3%	***	99
Dim Reduction	PCA	SVM_SIG	8.43e-05	+0.0%	***	99
Dim Reduction	LDA	SVM_SIG	1.55e-01	+3.3%		99
Dim Reduction	PCA	RF	1.92e-01	+0.0%		99
Dim Reduction	LDA	RF	4.57e-01	+0.0%		99

4 RUNTIME PERFORMANCE: HPC VS LOCAL WORKSTATION

All pipeline configurations in our study were trained and evaluated on institute's HPC infrastructure (Mesocentre). The jobs were run on Dell PowerEdge C6420 nodes, each equipped with 32 CPU cores, powered by Intel® Xeon® Gold 6142 (SkyLake) processors running at 2.6 GHz. Each job was allocated one node, utilizing all 32 CPU cores, and the multiprocessing functionality was implemented in Python using the `multiprocessing` module to efficiently parallelize the workload. Jobs were submitted using the SLURM job scheduler.

We tested the feasibility of running these tasks on a local workstation by running some pipeline configurations locally. These runs took approximately 3x longer than on the HPC cluster. The local workstation used for comparison featured an Intel® Core™ i7-1365U processor (12 threads), 32 GB RAM, and was running 64-bit Ubuntu 22.04.5 LTS. As an example, running the pipeline with the following configuration:

- Classifier: MLR_L2.
- Row Scaling: `StandardScaler`
- Col Scaling: `StandardScaler`
- Feature Selection: None
- Dimensionality Reduction: None
- Hyperparameter Tuning: Random Search

Note that the above pipeline configuration corresponds to that employed in section 3.3 of the Results. This required 1086 seconds on the local workstation, while it completed within 368 seconds on the HPC cluster; a difference of 2.95x. This indicates the feasibility of running these jobs locally, if required, but demanding longer run times for completion. This can be an important factor when exploring a large number of pipeline configurations as we have attempted here.
